# AIDS Cholangiopathy in an Asymptomatic, Previously Undiagnosed Late-Stage HIV-Positive Patient from Kenya

**DOI:** 10.4061/2011/465895

**Published:** 2011-04-04

**Authors:** Yiming Gao, Kathryn Chin, Yehia Y. Mishriki

**Affiliations:** ^1^Lehigh Valley Health Network, Allentown, P.O. Box 689, PA 18105-1556, USA; ^2^Penn University College of Medicine, 500 College Drive, Hershey, PA 17033, USA

## Abstract

AIDS-associated cholangiopathy is a form of biliary tract inflammation with stricture formation seen in AIDS patients who are severely immunosuppressed. It is no longer common in countries in which HAART therapy is widely employed but is still seen in underdeveloped countries. The majority of patients are symptomatic at the time of presentation. Herein, we describe a seventy-four-year-old woman who presented with unilateral leg swelling after a prolonged airplane flight. She was otherwise entirely asymptomatic. Routine laboratory testing was notable for a hypochromic microcytic anemia, slight leukopenia, and mild hypoalbuminemia. Liver enzymes were all elevated. Deep venous thrombosis was confirmed, and a CT scan of the chest disclosed no pulmonary emboli. However, the visualized portion of the abdomen showed dilatation of the common bile and pancreatic ducts. This was confirmed on ultrasonography and MRCP, and no obstructive lesions were noted. An ERCP revealed a dilated common bile duct without filling defects or strictures. A balloon occlusion cholangiogram showed strictures and beading of the intrahepatic ducts. Shortly thereafter, serology for HIV returned positive along with a depressed CD4 cell count, and the patient was diagnosed with AIDS-associated cholangiography.

## 1. Case Presentation

A seventy-four-year-old Kenyan woman was admitted to the hospital with bilateral lower extremity edema. Three days earlier, she had arrived to the United States after a sixteen hour flight. Past medical history included a stroke with no residual deficits and chronic low-back pain. Her family history was unremarkable. The patient had a 30 pack-year history of smoking but had quit 3 years earlier. She neither drank nor used illicit drugs. Review of systems was notable for generalized pruritus without rash, a periodic nonproductive cough, dysphagia with globus sensation, and a recent unintended weight loss of 14 kg. She had not been sexually active in more than thirty years. 

Her vital signs included a temperature of 36°C, blood pressure 104/68 mmHg, respiratory rate of 16 per minute, heart rate 80 per minute, and O_2_ saturation 98% on room air. The initial physical exam was only remarkable for 2+ edema in the left lower extremity as well as bilaterally diminished lung sounds with occasional faint late inspiratory crackles at the bases. 

Abnormal “routine” laboratory tests included hemoglobin 99 g/L, hematocrit 0.32 vol RBC/vol whole blood, WBC count 3,400/cc, 8% eosinophils, MCV 66 fL, and albumin 32 g/L; liver enzyme panel revealed an AST of 79 U/L (normal: 7–40 U/L), ALT of 67 U/L (normal: 7–40 U/L), alkaline phosphatase of 252 U/L (normal: 30–136 U/L), and GGTP of 287 U/L (normal: 8–78 U/L). The bilirubin and prothrombin time were normal. A venous duplex of the lower extremities revealed a thrombus in the left superficial femoral vein extending into the left popliteal vein. She was placed on heparin and warfarin.

An initial chest X-ray evealed a right lower lobe patchy infiltrate versus atelectasis. Given her history of cough and weight loss, the patient was treated with ceftriaxone and azithromycin for possible pneumonia and placed on droplet precautions while a PPD was placed and a QuantiFERON Gold lab test was obtained. Written consent was also obtained to complete an HIV test. 

**Figure 1:**
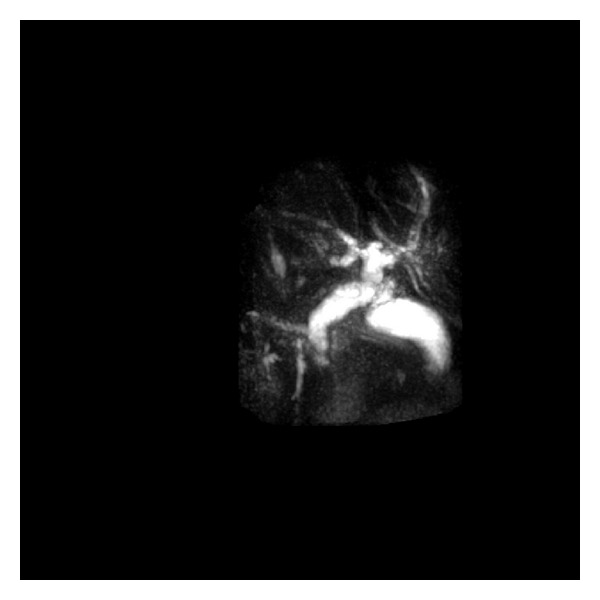


**Figure 2:**
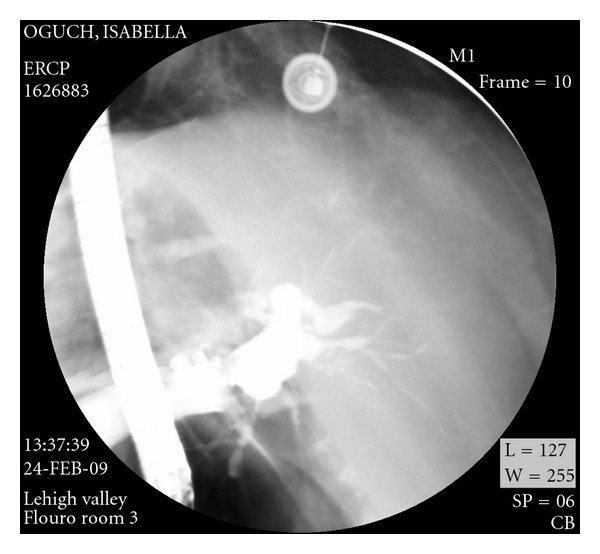


A CT scan of the chest showed no significant pulmonary pathology or embolism. The visualized portion of the abdomen showed dilatation of the common bile and pancreatic ducts. An abdominal ultrasound later confirmed dilatation of the common bile duct and showed central intrahepatic duct dilatation as well. No stones were visualized, but the distal portion of the common bile duct was obscured by overlying bowel gas. Magnetic resonance cholangiopancreatography (MRCP) showed pancreatic and biliary ductal dilatation without identification of any obstructive lesions. (Figure 1). An ERCP revealed a dilated common bile duct without filling defects or strictures. It was, however, very difficult to fill up the intrahepatic ducts. A 15 mm balloon occlusion cholangiogram was, therefore, performed and showed strictures and beading of the intrahepatic ducts. (Figure 2) The patient was felt to have sclerosing cholangitis, and was started on ursodiol 300 mg three times daily. 

Shortly thereafter, the patient's HIV test came back positive. The CD count was 124 per cubic millimeter with an HIV viral load of 2.6 million copies/mL. As it was suspected that the patient suffered from AIDS cholangiopathy, further laboratory studies were obtained. A stool study for Isospora ova and parasites and a direct stool study for Cyclospora were negative. An electroimmunofluroscent assay for cryptospora was also negative. The IgM level of CMV antibody was 0.34 (nl: <0.91 antibody index; equivocal 0.91–1.09; positive >1.09), but the IgG level of CMV antibody was elevated at 4.74 antibody index.

## 2. Discussion

The term “AIDS cholangiopathy” (aka AIDS-related cholangitis) was first coined in 1986 by Margulis et al. who described biliary tract abnormalities in patients with AIDS [1]. However, in 1983, Guarda et al. had previously described two cases of cryptosporidiosis in patients with the acquired immune deficiency syndrome, one of whom had gallbladder involvement [2]. That same year, Pitlik et al. described two patients with biliary papillary stenosis who suffered from cryptosporidiosis [3]. In early studies, AIDS cholangiopathy was estimated to occur in up to 26 percent of AIDS patients, but its true incidence is unknown as many patients are asymptomatic. No prevalence studies have been published, but in the 1990s the prevalence was estimated to be as high as 30% in patients with chronic AIDS-related diarrhea [4]. Since the advent of highly active antiretroviral therapy (HAART) it has become less common in the United States, but in areas with limited access to healthcare such as Kenya, it may be as high as 45% [5]. AIDS cholangiopathy is usually seen in patients with CD4 counts below 100/mm^3^ [6]. It usually presents in patients with a known diagnosis of HIV, but it can rarely present as an AIDS-defining illness [7].

The etiology of AIDS cholangiopathy is unknown, but it is believed to be related to opportunistic infections in the biliary tract where pathogens have often been detected. These infections likely cause a secondary sclerosing cholangitis due to the associated continual inflammation. It is theorized that enteric infection leads to portal bacteremia which, in turn, leads to subsequent bile duct injury and destruction [8]. HIV itself may play a role in some manifestations of AIDS cholangiopathy. However, although it has been isolated in the bowel mucosa, it has never been detected in the biliary epithelium. A genetic predisposition has been postulated as pathogens may trigger an autoimmune-mediated reaction in patients with HLA DRw52a [8]. This may explain why the features of AIDS cholangiopathy are independent of the associated opportunistic infection [9].

The pathogens associated with AIDS cholangiopathy are listed in Table 1. AIDS cholangiopathy is usually associated with bacterial or Mycobacterium tuberculosis infection when the CD4 count ranges from 200 to 750 cells per cubic mm. With CD4 counts less than 200 cells per cubic mm, associated infections are viral, protozoan or fungal, or caused by Mycobacterium avium intracellulare [10]. The two most commonly detected pathogens are Cryptosporidium and cytomegalovirus (CMV). When there is involvement of the intrahepatic ducts, Cryptosporidium and CMV infection are more likely than when the intrahepatic ducts are not involved [10]. 

Cryptosporidium was the first opportunistic infection reported in the bile ducts of AIDS patients in 1983 by two independent groups, Pitlik et al. [3] and Benhamou et al. [11]. It is also a common opportunistic infection in AIDS cholangiopathy detected in 6% of AIDS patients in the pre-HAART era and in 21% of those with diarrhea [12]. During the cryptosporidiosis outbreak in Milwaukee's municipal water supply in 1993, four-hundred thousand people were exposed to cryptosporidiosis, and 29% of AIDS patients that developed cryptosporidiosis had biliary tract involvement. AIDS patients with CD4 counts less than 50 per cubic millimeter were more likely to have biliary symptoms and an increased risk of death at one year [13]. Cryptosporidium is associated with severe and diffuse changes in the biliary tract. It initiates an inflammatory response that is mostly composed of lymphocytes with occasional plasma cells and macrophages and rare eosinophils. Neutrophils can be seen in the lumina of dilated glands forming microabscesses. The cells of the biliary epithelium often have enlarged nuclei with an eosinophilic cytoplasm and are transformed into cuboidal or attenuated cells [14]. Squamous metaplasia can also occur [15]. Polypoid defects have been hypothesized to be caused by granulation tissue that extends into the bile ducts [16]. Cryptosporidium parvum has also been linked to the development of sclerosing cholangitis in other immunocompromised patients such as those with X-linked hyper IgM syndrome [5]. 

Before the introduction of HAART therapy, cytomegalovirus was detected in up to two-thirds of autopsies in AIDS patients. These patients often had coinfection with Cryptosporidium and Candida albicans. Common bile duct dilatation and a mural thickening of an acalculous gallbladder were often noted [5]. It has been suggested that cytomegalovirus could lead to ischemic injury of the biliary tract in a fashion similar to that in posttransplant patients and patients undergoing intraarterial chemotherapy who develop bile duct injury that resembles sclerosing cholangitis. The inclusion bodies that are characteristic of cytomegalovirus infection are rarely seen in biliary epithelial cells of affected patients, but are frequently found in the arteriole adjacent to the biliary duct [17]. Often there is an associated lymphocytic infiltrate [7]. Viral cultures for CMV are usually negative but immunohistochemical stains may improve CMV detection [6]. In situ DNA hybridization is the method of choice for detecting CMV in tissue samples [18]. Microsporidia is uncommon in patients with AIDS cholangiopathy. In a study by Ko et al., it was not identified in any of the patients with AIDS cholangiopathy [19]. However, there have been other reports that identified the organism although in fewer than 10% of patients [6]. It had been suggested that patients with no identified pathogen may have microsporidial infection as multiple biopsies are often required for its detection. *Enterocytozoon bieneusi* can be detected with hematoxylin and eosin stain, but is more apparent with Giemsa stain. In some cases, electron microscopy may be necessary [6, 20]. Isospora and Mycobacterium avium intracellulare (MAI) are less common causes of AIDS cholangiopathy contributing to fewer than 5% of cases [7]. Mycobacterium avium complex is a consideration when one sees poorly formed noncaseating granulomas along with large foamy macrophages and a scarcity of lymphocytes [21]. Malignancies such as Kaposi's sarcoma or primary Burkitt's lymphoma can also cause AIDS cholangiopathy, but this is far less common than an infectious etiology [7, 21]. Finally, diseases of the pancreas that involve the distal common bile duct can mimic AIDS cholangiopathy [21].

Affected patients often present with right upper quadrant abdominal pain (99%) and, less commonly, with fever and jaundice (10%) [4]. Abdominal pain is typically more severe when papillary stenosis is present [5]. It is characteristically sharp in nature and may radiate to the back. Nausea and vomiting are not uncommon [22]. Fever occurs in over half of patients with AIDS cholangiopathy, and high spiking fevers can indicate a bacterial superinfection [7]. Patients who are infected with cryptosporidium, microsporidium or MAI additionally may have diarrhea and malabsorption symptoms [7, 22].

Laboratory examination is helpful but not specific in the diagnosis of AIDS cholangiopathy. The classic laboratory profile in AIDS cholangiopathy is an alkaline phosphatase that is five- to seven-times the upper limit of normal and a moderate increase in transaminase levels. Alkaline phosphatase elevation may be the sole laboratory abnormality in some patients. Jaundice in AIDS cholangiopathy is usually mild with a total bilirubin less than twice the upper limit of normal indicating incomplete obstruction. In approximately 20% of patients with AIDS cholangiopathy, however, all aforementioned lab tests are within normal limits even with diagnostic findings on ERCP [5]. Amylase levels may be elevated in 10% of patients [7].

Ultrasonography is useful in the initial screening of AIDS cholangiopathy as it can provide the intraluminal caliber, thickness of the gallbladder wall and bile ducts, the presence of stones, sludge and/or pericholecystic fluid [23]. It is abnormal in approximately 75% of patients with AIDS cholangiopathy [7]. Earlier studies indicated that up to 25% patients with AIDS cholangiopathy have normal ultrasounds especially if the disease is isolated to the intrahepatic ducts [21], but larger more recent studies suggest that ERCP adds little benefit, as ultrasound has a sensitivity of 97% and a specificity of 100% [24]. Endoscopic ultrasound allows for better detection of dilation and thickening of the common bile duct and can more accurately exclude stones, extrabiliary compression, and tumors when compared to transabdominal ultrasound [11].

CT is more useful than ultrasound in identifying intrahepatic strictures [7]. It is also better at disclosing abnormalities of the pancreas and liver [9]. However, it is less successful than ultrasound in demonstrating wall thickening and strictures of the common bile duct [7]. Radionuclide hepatobiliary scanning (HIDA) can detect duct dilatation but has been supplanted by other imaging modalities. It may be a useful in patients suspected of having acute cholecystitis. Of note is the fact that 20% of patients will have normal noninvasive imaging studies [4]. The gold standard for diagnosing AIDS cholangiopathy is by direct visualization of the biliary tracts via ERCP [8]. Biliary brushings or aspirated biliary fluid may help make the diagnosis of Cryptosporidium or Microsporidia. ERCP is useful in obtaining biopsy samples and for testing for CMV, Cryptosporidium, and Mycoplasma. It is also particularly useful in patients with severe pain, allowing for sphincterotomy. ERCP has not been recommended in patients with asymptomatic AIDS cholangiopathy [21].

Four subcategories of AIDS cholangiopathy have been described: (1) papillary stenosis (15%), (2) intrahepatic sclerosing cholangitis-like lesions (20%), (3) a combination of papillary stenosis and intrahepatic sclerosing lesions (50%), and (4) long extrahepatic bile duct strictures with or without intrahepatic lesions (15%) [4]. Papillary stenosis is defined as a common bile duct diameter of >8 mm with tapering of the distal 2–4 mm of the common bile duct with marked retention of contrast beyond 30 minutes. It is unknown if the different subtypes are a continuum of one disease process [21]. The common bile duct can have a beaded or scalloped appearance. The left intrahepatic ductal system is more severely involved than the right side in most cases. In up to 25% of cases, intraluminal polypoid defects are seen in the common bile duct and/or the large intrahepatic strictures [7]. The combination of papillary stenosis and intrahepatic ductal strictures is relatively unique to AIDS cholangiopathy [5]. In HIV-infected individuals, the presence of an echogenic nodule in the distal common bile duct may indicate AIDS cholangiopathy, as it is thought to represent edema in the papilla of Vater [25].

The intrahepatic findings in AIDS cholangiopathy are comparable to those seen in primary sclerosing cholangitis (PSC) with a beaded appearance and a diminished arborization or pruning of peripheral branches. However, the extrahepatic manifestations of AIDS cholangiopathy are quite different from PSC (Table 2). Diverticular outpouchings, sacculations, and high-grade proximal strictures are seen in PSC while moderate ductal dilatation with irregular margins and nodules are more characteristic of AIDS cholangiopathy [9]. Isolated dilatation of intrahepatic biliary ducts in AIDS cholangiopathy (i.e., Caroli disease) is very uncommon but has been reported [8]. MRCP may be an alternative for patients with high CD4 counts that are receiving HAART therapy as they do not require biopsy specimens to test for opportunistic infections [18]. In our patient, MRCP showed a dilated common bile duct and subtle irregularities of the intrahepatic ducts. In general, percutaneous liver biopsy has little value in diagnosing AIDS cholangiopathy [21, 26].

In addition to imaging features, biopsy will often show an inflammatory infiltrate that is rich in T4 lymphocytes in primary sclersoing cholangitis while these cells are depleted in AIDS patients. The characteristic finding on biopsy in AIDS cholangiopathy is prominent mucosal folds which indicate inflammation and edema. This mucosal thickening can be diffuse or it can be focal in which case it is fine and nodular [9]. Approximately 26% of patients with AIDS cholangiopathy have AIDS-related polypoid cholangitis which consists of intraluminal polypoid defects within the common bile duct and the larger intrahepatic ducts. Histologically, the polypoid defects correlate with the presence of granulation tissue and do not affect Prognosis [16].

Treatment of AIDS cholangiopathy is primarily endoscopic sphincterotomy for papillary stenosis and stenting for dominant common bile duct strictures. Sphincterotomy provides persistent pain relief, but unfortunately has not been shown to prolong survival or improve transaminase elevation [5]. Alkaline phosphatase levels continue to rise as intrahepatic bile duct sclerosis progresses [20]. 21 One option for pain that is refractory to endoscopic therapy is CT-guided celiac plexus blockade [5]. For patients with intrahepatic and extrahepatic sclerosing cholangitis treatment options are limited. Although AIDS cholangiopathy is most commonly caused by opportunistic infections, medical treatment against C. Parvum, CMV, or microsporidium does not alter symptoms or anatomical abnormalities already present [5]. However, it is still important to treat patients for any detected infections to prevent nonhepatic complications such as gastrointestinal symptoms from Cryptosporidium or retinitis from Cytomegalovirus. Many patients also have improvement in symptoms after initiation of antiretroviral therapy [27]. 

Ursodeoxycholic acid has been used in patients with AIDS cholangiopathy, given that the intrahepatic changes are similar to those found in primary sclerosing cholangitis. It has been shown to improve liver biochemistry. The recommended dose is 300 mg three times daily primarily for patients with intrahepatic ductal disease and elevated liver function tests. Patients with pain refractory to ECP may experience relief with ursodeoxycholic acid [28]. 

In the pre-HAART era, the survival of patient with AIDS cholangiopathy had been generally dictated by the natural course of AIDS rather than the cholangiopathy since this disease tends to occur in patients with advanced AIDS. AIDS cholangiopathy was, itself, rarely fatal as other AIDS-related illnesses were usually the cause of death in these patients. In patients diagnosed with AIDS cholangiopathy before the introduction of HAART, the median survival time was 5–8 months compared to the 14 months median survival for those diagnosed with AIDS cholangiopathy after 1996 [19]. CD4 lymphocyte counts, duration on HIV seropositivity, and the patient's age had previously been reported as prognostic factors for AIDS cholangiopathy [13, 19, 29]. However, Ko et al. identified two variables linked to poor prognosis. One was the presence or history of any opportunistic infection of the GI tract, lung, eye, nervous system, skin, or of systemic involvement (hazard ratio of 3.24) or cryptosporidial infection (harzard ratio of 2.69.) The other risk factor was a high alkaline phosphatase level of either >1000 IU/L or eight times above the upper limit of normal (hazard ratio of 2.69). In their study, CD4 counts, type of cholangiopathy, and sphincterotomy did not affect life expectancy. They also suggested HIV viral loads as a possible prognostic factor, but this was unavailable for most patients in their study [19].

Finally, it is recommended that patients with AIDS cholangiopathy intermittently have abdominal ultrasonography and CA 19-9 levels screening for cholangiocarcinoma. An early diagnosis might allow for complete resection of the tumor [30]. 

## Figures and Tables

**Table 1:** Infectious causes of AIDS-associated cholangiopathy.

Bacteria, Mycobacteria	Viruses	Protozoa	Fungi
Mycobacterium avium-intracellulare Mycobacterium kansasii Mycobacterium tuberculosis Rochalimaea henselae Rochalimaea quintana Salmonella enteritidis Salmonella typhimurium Enterobacter cloacae Campylobacter fetus	Cytomegalovirus Herpes simplex Adenovirus HIV	Pneumocystis carinii Microsporidia Enterocytozoon bieneusi Encephalitozoon cuniculi Encephalitozoon intestinalis Cryptosporidium parvum Leishmania donovani Toxoplasma gondii Dicrocoelium dendriticum Cyclospora cayetanensis Isospora	Histoplasma capsulatum Cryptococcus neoformans Coccidioides immitis Candida albicans

**Table 2:** Imaging features of AIDS cholangiopathy versus primary sclerosing cholangitis.

	AIDS cholangiopathy	Primary sclerosing cholangitis
Dilation common bile duct	Common	Rare
Intrahepatic ducts	Affected but usually normal in number	Significantly decreased in number
Intraductal debris	Common	Rare
Intraductal filling defects	Yes	None
String-like strictures	Rare	Common
Diverticular outpouchings and sacculations	Rare	Common
Papillary stenosis + intrahepatic ductal stricture	Common	Never reported
Extrahepatic stricture morphology	Irregular margins and nodules reflecting an often focal nature	Irregular strictures with entire ducts involved
Gallbladder wall thickening	Common	Rare
